# Synchronization in Singing Duo Performances: The Roles of Visual Contact and Leadership Instruction

**DOI:** 10.3389/fpsyg.2018.01208

**Published:** 2018-07-17

**Authors:** Sara D'Amario, Helena Daffern, Freya Bailes

**Affiliations:** ^1^Department of Electronic Engineering, University of York, York, United Kingdom; ^2^School of Music, University of Leeds, Leeds, United Kingdom

**Keywords:** timing, synchronization, ensemble performance, visual contact, leadership

## Abstract

Interpersonal synchronization between musicians during ensemble performances is characterized by continuous micro-timing adjustments due to intentional and unintentional factors supporting expressive interpretations, or caused by noise during the cognitive-motor process. Whether visual contact between musicians and the instruction to act as leader or follower affect synchronization in ensembles remains mostly unclear. This study investigates the role of visual cues and leader-follower relationships in singing performances. Twelve vocal duos took part in the study, singing a two-part piece, which was composed for the study and was mostly homophonic in structure. Four conditions were applied in a randomized order: with and without visual contact, and with a designated leader or follower. The piece was repeated four times in each condition, and the condition presented three times, for a total of 12 performances of the piece in each condition. Data were acquired using electrolaryngograph electrodes and head mounted microphones to track the fundamental frequency estimates of the individual singers. Results show that the presence and absence of visual contact had a significant effect on the precision and consistency of synchronization during singing duo performances. Precision and consistency were better in the presence of visual contact between singers than without, and these effects were associated with the beginning of phonation of the first note of the piece. The presence/absence of visual contact also had an effect on the tendency to lead or lag a co-performer associated with the onset of the first note; the extent of leading was greater when visual contact was absent. The instruction to act as leader or follower did not affect precision or consistency of synchronization, nor did it relate to the observed tendency to precede or lag a co-performer. The results contribute to the tailoring of rehearsal strategies, as singers and directors can be better informed of the factors influencing synchronization and focus on specific areas of difficulty in certain performance conditions, such as first note onsets when performers are not able to see each other.

## Introduction

Timing within a music ensemble performance varies within and between players, establishing small asynchronies between members of an ensemble. This variability in Western Classical music is mostly intentional and pre-planned, relating to the musical score or shared intentional deviations from the score in support of expressive goals, such as deliberately slowing the *tempo* at the end of the piece (Phillips-Silver and Keller, 2012) or delaying some notes as a means of emphasis. A certain amount of this variability is unintentional, due to technical and/or expressive complexity and noise during the cognitive-motor processes (Ragert et al., 2013). Musicians generally try to limit and control the extent of these inter-performer temporal fluctuations through individual practice and collaborative group rehearsals, with the purpose of establishing shared performance goals based on knowledge of the musical structure and the playing style and expressive intentions of the co-performer(s) (Williamon and Davidson, 2002; Ginsborg et al., 2006).

The variability in note onset asynchronies between performers in professional ensembles, when playing between 40 and 130 beats per minute (bpm), is typically very small, in the order of tens of milliseconds, and decreases with increasing tempo (Rasch, 1979, 1998). Standard deviation values of 24 and 28 ms were measured for asynchronies in string quartets playing at 157 bpm (Wing et al., 2014b). Such high levels of coordination are maintained through iterative temporal adjustments: people may adapt the timing of their finger tapping to that of an autonomous timing source such as a metronome in tapping tasks (Repp and Su, 2013); musicians may correct the tempo to one of the co-performers or each player may adjust the tempo for the temporal fluctuations of the other (Goebl and Palmer, 2009).

A number of factors can affect temporal synchronization. Recent research conducted with piano duos shows that interpersonal coordination is influenced by the complexity of the piece being played, the auditory feedback from co-performer(s), the familiarity with co-performers' playing styles, and the musicians' levels of ensemble expertise (Keller et al., 2007; Goebl and Palmer, 2009; Keller and Appel, 2010; Loehr et al., 2011).

Visual contact between members of an ensemble is also a key element that can affect temporal synchronization in joint music performance. Investigations of unintentional interpersonal communication in non-musical contexts have demonstrated an effect of visual contact on interpersonal entrainment (Oullier et al., 2008). Studies analyzing the role of visual contact in musical scenarios have demonstrated that eye contact is often used in popular music bands (Kurosawa and Davidson, 2005); and, performers have been reported to look at a videotaped conductor for 28% of the performance duration (Fredrickson, 1994). It has also been found that the frequency of visual contact among string quartet players did not change in relation to the stress associated with the performance setting, i.e. rehearsal setting vs. public recital (Biasutti et al., 2016). A number of studies have revealed that visual cues improve the communication of interpersonal intentions between musicians (Dahl and Friberg, 2007; Castellano et al., 2008). Qualitative investigations have suggested that eye contact also improves synchronization in musical ensembles (Williamon and Davidson, 2002; Clayton, 2007).

A few quantitative studies analyzing the benefits of visual contact for temporal synchronization in the music ensemble context have elicited complex results. Keller and Appel (2010) found that the presence or removal of eye contact did not markedly affect synchronization between pianists in duo performances, as indexed by the median of signed and unsigned asynchronies, calculated by subtracting the onset times of the *primo* part from the *secondo* part. However, higher variability of temporal synchronization, as indexed by the coefficient of variation (CV) of signed asynchronies, was found in the presence of visual contact compared with when visual contact was removed, which the researchers speculated could be because the musicians may have focused more on expressive timing variation. Research also suggested that visual cues between pianists are more important when auditory feedback is limited compared with full auditory feedback (Goebl and Palmer, 2009), and that eye contact might be important for the temporal coordination between pianists (Kawase, 2014). The different results reported by Keller and Appel (2010); Goebl and Palmer (2009), and Kawase (2014) might be explained in relation to the characteristics of the musical stimuli being performed, as discussed by Bishop and Goebl (2015). In the first two studies, the authors made use of pieces with a regular meter, while the latter utilized a rhythmically complex piece featuring tempo changes and long pauses. The effect of visual contact in relation to tempo change was tested by Bishop and Goebl (2015), demonstrating that eye cues positively affect temporal synchronization during piano-piano duo and piano-violin duo performances, when following long pauses in the music. These results suggest that visual contact between pianists or piano-violin players might come into play as a secondary support in improving synchronization when auditory feedback is limited or musical timing is irregular.

However, there has been very little study on the effect of visual contact between singers during ensemble performances to date. A recent case study conducted by D'Amario et al. (2018) with two semi-professional singing duos suggests that controlling visual contact might affect synchronization between musicians. As the study highlights, the effect of the presence or absence of visual contact between musicians might apply only to note beginnings, and not note endings, and interpersonal synchronization temporally computed at note beginnings might be different from that observed at note endings. These findings suggest that the analysis of synchronization in vocal ensembles should not be limited to the onsets as it is in most of the literature analyzing instrumental ensemble performances, but should also take into account the degree of synchronization at note endings. Coordination at note endings may not be a meaningful measure for pianists given their use of the damper pedal, but might be an important measure for most other types of music ensembles, including singing ensembles, where musicians do try to synchronize offsets as well as onsets, as the tight offset asynchronies reported in D'Amario et al. (2018) suggest. However, the small sample size of this preliminary investigation prevents any general conclusions from being drawn. Further investigations are needed to understand the effect of visual contact on synchronization between singers during vocal ensemble performances. For example, the degree of synchronization might be greater at the beginning of phonation compared with the synchronization of other note beginnings within a *legato* phrase; singers might find it harder to be together, when there is no previous temporal reference.

Synchronization in joint music performance may also be influenced by group roles such as leader-follower relationships between members of a musical ensemble. Loehr et al. (2011) found that pianists playing the left-hand accompaniment tended to anticipate the onsets of the upper melody played by another pianist during piano duet performances. A number of case studies have recently investigated leadership in string quartet performances by analyzing body movements (e.g., head and instrument's bow) in relation to acoustic cues. Timmers et al. (2013) and Timmers et al. (2014) show a complex pattern of relationships between musicians during string quartet performances, rather than a traditional role division of leadership characterized by the artistic attribution of leader to the first Violin, whilst the co-performers play organizational, social roles or act as a co-leader (King, 2006). Glowinski et al. (2012) demonstrated the relative leadership of the first violin, investigated through the analysis of the movements of the musicians' heads toward a common point of reference. Results show that the first violin exhibited the highest number of driving forces, an indicator of the relative importance of the musician, although that of the other musicians remained close to the first violin. A study conducted by Badino et al. (2014) tried to force the unidirectional communication between the first Violin of a string quartet and the co-performers, by applying temporal and dynamic changes to the score, known only to the first Violin, across repeated performances. Results show that when perturbations were introduced, unidirectional influence from the leader decreased, suggesting that leadership might depend on the sharing of knowledge between performers.

Leader-follower relationships have also been investigated by assigning specific group roles. Goebl and Palmer (2009) found that pianists performing the melody part of a piano duet piece and instructed to act as the leader and determine the tempo, tended to precede the onsets of the other pianist playing the accompaniment part and acting as the follower. Zamm et al. (2015) further analyzed synchronization in piano duets, showing a compensatory timing behavior between pairs of pianists performing the same melodies in a round, characterized by a delay in temporal attack between one pianist who begins and is assigned to the role of leader, and a second pianist who enters later and is assigned the role of follower. The study reports, in fact, that the followers' onsets precede those of the leader, showing a directionality that is opposite to the researcher's instructions and to the musical structure. Although the analysis was not able to identify whether this directionality was due to the follower striving to catch up, or to the leader lagging behind, a compensatory behavior is evident. The contrasting results highlighted by Goebl and Palmer (2009) and Zamm et al. (2015) regarding the amount of leadership exhibited by the designated leaders and followers might be caused by the different music material used for the experiment. In the former, pianists played three two-part pieces with different melodies and note-ratios between the parts; in the latter, participants performed the same parts in unison and in round. Furthermore, researchers' requirements to keep a fixed tempo by listening to a metronome before each trial, and to instruct the designated leader to be responsible for determining the tempo, might have had an effect on the leader-follower relationships.

Recently, the case study conducted by D'Amario et al. (2018) further investigated leadership by analyzing synchronization that spontaneously emerges in two singing duos without instructions to focus on timing. This preliminary investigation highlighted bidirectional temporal adaptation between singers in vocal duo performances and suggests that instructing singers to act as leader or follower, but without controlling for timing with a metronome or instructing them to focus on synchronization, might affect the tendency to precede or lag a co-performer at note beginnings. The study also found that leadership instruction had a significant effect on the consistency of synchronization between singers, although in different ways across duos: when the upper voice was assigned the role of leader, consistency of note beginning asynchronies, as indexed by SD and CV of absolute asynchronies, was significantly worse in one duo, but better in the other, suggesting the need for further investigation. The restricted data set collected from only two singing duos prohibits any generalizable results and illustrates the need for further investigations in this field of research.

Although leader-follower roles are generally conceptualized as social roles, rather than in terms of performing timings, the above findings overall suggest that investigating the anticipation-delay of onsets and note beginnings by performers within an ensemble is a valuable indicator of group roles. The studies conducted so far to understand music roles through the analysis of synchronization between musicians during ensemble performances have also highlighted the complexity of the phenomenon and the need for future investigations. For example, the effect of the instruction to act as leader or follower without a focus on time-keeping or leadership clearly induced by the score is not fully understood. Investigation to this end would be particularly beneficial for singing ensembles, since the literature analyzing temporal coordination has been mostly focussed on instrumental ensembles. Moreover, research should be conducted analyzing behaviors at note beginnings and endings.

In summary, research suggests that synchronization in instrumental ensembles might be affected by group roles such as leader-follower relationships and visual contact between musicians. However, the effect of visual contact between musicians and the instruction to act as leader or follower on the interpersonal synchronization between singers during vocal ensemble performances has not yet been fully investigated. The current study aims to investigate the roles of altered visual contact and leadership instruction on synchronization during ensemble singing, addressing the following questions:

- Do visual contact and acting as leader or follower affect synchronization between singers in vocal duos?
- What are the differences in synchronization patterns between onsets, offsets, and note beginnings and endings?
- Are these differences affected by visual contact and leadership instruction and/or associated with the beginning of phonation ?

Although this study is exploratory in nature, it was hypothesized that the degree of synchronization is better with visual contact between singers than without. Previous investigations (Goebl and Palmer, 2009; Zamm et al., 2015; D'Amario et al., 2018) did not report conclusive findings regarding the effect of leadership instruction, but apparently contrasting results that the researchers speculate relate to the score. For this reason, there was no specific hypothesis to test in the study, which was mainly an observational investigation of the leadership instruction. Nevertheless, research conjectured that instruction to act as leader or follower affects synchronization between singers, based on previous evidence regarding singing ensembles. It was also hypothesized that these effects change in relation to note beginnings and endings, when musicians perform regular rhythms with no tempo change, as found in D'Amario et al. (2018).

## Method

### Participants

Ethical approval for the study was obtained from the Physical Sciences Ethics Committee (PSEC) at the University of York (UK) with reference D'Amario151127. Twenty-four singing students from the Department of Music at the University of York participated in the current experiment (14 female, age *M* = 20.9, *SD* = 2.9). Twenty of them were undergraduate students, and four of them postgraduate students with singing as first study. They had at least 3 years' formal singing practice (*M* = 8.6, *SD* = 4.5) and at least 5 years' experience performing in a singing ensemble (*M* = 10, *SD* = 5.7), but they had not sung together prior to the experiment. They reported having normal hearing and not having absolute pitch.

### Stimulus

**Figure 1:**
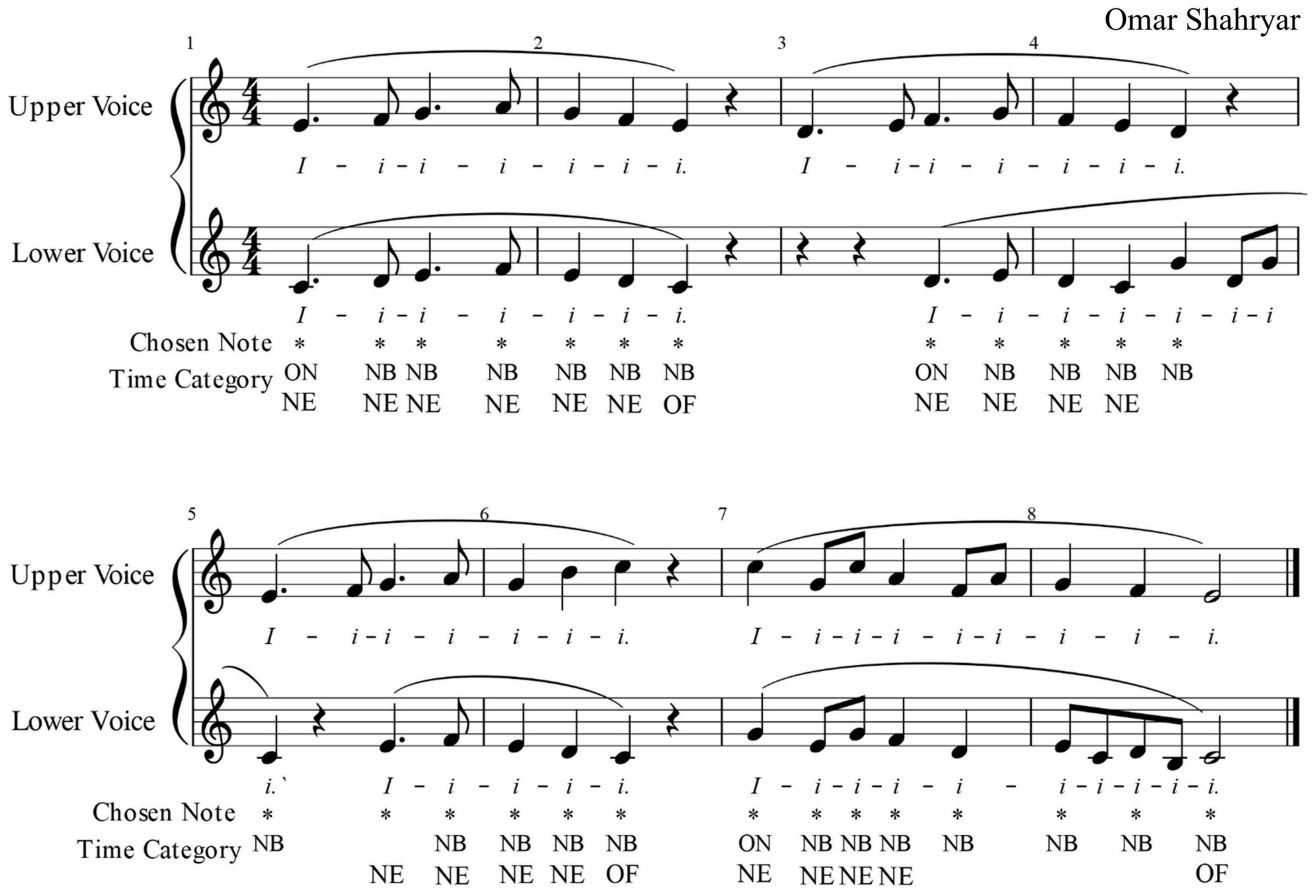
Duet exercise from a previous study (D'Amario et al., 2018) that was used in the present investigation, showing the notes chosen for the analysis and the four sets of time categories (e.g., ON, onset; NB, note beginning; NE, note ending; and OF, offset). The figure is ©the authors, licensed CC-BY.

This study made used of a vocal duo exercise that was composed for a previous case study, D'Amario et al. (2018), featuring mostly a homophonic texture that facilitates analysis of synchronization, as shown in Figure 1. The upper voice (UpperV) was assigned a higher tessitura than the lower voice (LowerV).

### Apparatus

The experiment took place in a recording studio at the University of York, treated with absorptive acoustic material. Audio data were collected using head-mounted close proximity microphones (DPA 4065), placed on the cheek at approximately 2.5 cm from the lips, and a stereo condenser microphone (Rode NT4) placed at equal distance in front of the singers at approximately 1.5 m from the lips. In addition, electrolaryngograph recordings were collected using electrolaryngograph electrodes (Lx) from Laryngograph Ltd. (www.laryngograph.com) placed on the neck. Lx, widely used for the analysis of singing voice (Fourcin and Abberton, 1971; D'Amario and Daffern, 2017), was chosen because it allowed individual fundamental frequency analysis for each singer based on vocal fold activity rather than microphone recordings. The 6 outputs (2 Lx, head-mounted mics, 1 stereo mic comprising right and left channel) were connected to a multichannel hard disk recorder (Tascam DR680) and recorded at a sampling frequency of 48 kHz and 32-bit depth.

### Design

A total of four conditions were applied in a randomized order, as follows:

- VC_UpperVoiceL: with visual contact (VC), and upper voice designated Leader and lower voice Follower (UpperVoiceL)
- VC_UpperVoiceF: with visual contact (VC), and upper voice designated Follower and lower voice Leader (UpperVoiceF)
- NVC_UpperVoiceL: without visual contact (NVC), and upper voice designated Leader and lower voice Follower (UpperVoiceL)
- NVC_ UpperVoiceF: without visual contact (NVC), and upper voice designated Follower and lower voice Leader (UpperVoiceF)

The piece was repeated four times in each condition, and each condition was presented three times. The study resulted in a 4 (conditions) × 3 (repeated performances of each condition), × 4 (repeated performances within each condition) design featuring a total of 48 repetitions of the piece per duo.

### Procedure

At the beginning of the session, singers received written and spoken instructions, and gave written informed consent. As reported in D'Amario et al. (2018), singers first practiced the piece together for 10 min, singing from the score to the vowel /i/, and listening for 10 s to a metronome set at 100 beats per minute (BPM) before starting to rehearse. At the end of the 10 min, if the singers were able to perform the piece by memory and without error, the four conditions were then presented; otherwise, they were invited to rehearse for 10 more minutes and then the test was repeated. Once the musicians passed the performance test without error, each singer was assigned the role of leader or follower according to the UpperVoiceL and UpperVoiceF conditions. Thus, in the former condition the upper voice was instructed to act as leader, and the lower voice as follower. These roles were reversed in the UpperVoiceF condition (i.e., the upper voice was instructed to follow, and the lower voice to lead). Signs labeled “Leader” and “Follower” were placed on the floor in front of the participants, to facilitate recalling of their role. Each singer only had one assigned part/musical voice. Musicians faced each other at a distance of 1.5 m in the visual condition and turned away from each other at the same distance in the non-visual contact condition. Participants were asked to sing at performance level and were unaware of the purpose of the study.

### Analysis

Two sets of data including the audio waveform from the head-mounted microphones and the Lx waveform were first imported into Praat as.wav files, and then *f*_*o*_ estimates extracted with a time step of 1 ms, as in D'Amario et al. (2018).

The analysis of interpersonal synchronization was conducted on the notes being relevant to synchronization, as shown in Figure 1. For each chosen note, a true starting and ending time stamp value was detected, based on the definition of the following 4 time categories, as in D'Amario et al. (2018):

- Onset (ON): beginning of phonation after a silence
- Note beginning (NB): beginning of a note within a *legato* phrase
- Note ending (NE): ending of a note within a *legato* phrase
- Offset (OF): ending of phonation followed by a silence

The extraction of the time categories was automated through the application of TIMEX, a peak picking algorithm that detects onsets and offsets of phonation and note beginnings and endings within a sung *legato* phrase from the acoustic and electrolaryngograph recordings. This algorithm, tested on a set of singing duo recordings, proved to be a *state-of-the- art* algorithm with an overall performance of 78% within a tolerance window of 50 ms compared with manual annotations performed by three experts in this field of research; it also proved to be a valuable and successful tool, recommended for investigations of interpersonal synchronization between singers (D'Amario et al., 2018). This event detection method was aurally and visually cross-validated for the entire data set by the first author. Soft phonation was specifically scrutinized in respect to the electrolaryngograph signal, which might not pick up very small vocal fold vibrations when the amplitude is very small. In cases whereby the phonation was too soft to be picked up by the Lx signal, the timing detection was mostly based on the acoustic recording. Pitch errors due to the musicians singing wrong notes were analyzed comparing the *f*_*o*_ values extracted and the acoustics and Lx recordings with the notated score. Notes in which a pitch error occurred were excluded from the analysis. The overall error rate was less than 1%.

Interpersonal asynchronies were then calculated to measure phase synchronization between singers, subtracting the follower's timestamp values from the leader's (leader minus follower) regarding each time category of the selected notes. Negative values indicate that the designated leader preceded the follower, while positive values mean that the follower was ahead of the leader as measured temporally at that specific time category and note.

Asynchronies that fell outside three times the interquartile range (IQR) were automatically identified as extreme outliers through SPSS (IBM SPSS Statistics v. 24) and excluded. The identification of outliers was run for each time category, performance condition and duo.

Three measures of synchronization were investigated, namely:

- *precision* of temporal synchronization, as indexed by the absolute asynchronies
- *consistency*, as indexed by the standard deviation (SD). This has been computed for each time category, note, condition, and duo, across the repeated performances within each condition. For example, SD asynchrony was computed for the onset of note 1 in Duo1 regarding the VC_UpperVoiceL condition across the 12 repeated performances featuring this time category/note/duo/condition.
- *tendency to precede or lag* a co-performer, as indexed by the signed asynchronies

To understand whether visual contact or leadership had an effect on synchronization, and whether the effects, if any, also depend on important voice entry points and/or time category, the analysis was run across the following three stages and levels:

- Stage 1–High level: considers the effect of the independent variables on the synchronization measures, incorporating the full data set.
- Stage 2–Medium level: investigates the effect of the independent variables at singers' simultaneous entries, based on the subset of data including note 1, 3, 19, and 22. Notes 1 and 19 were chosen as being points of simultaneous voice entry; whilst note 3 was selected to investigate whether any effect regarding the simultaneous entry at the beginning of the piece disappeared by the next downbeat (i.e., the third downbeat of bar 1, since note 1 is a dotted quarter); for similarity with bar 1, note 22 has been selected, being the third downbeat of that bar as well.
- Stage 3–Low level: analyses the effect of visual contact and leadership on the time category of those notes where a main effect was found at the medium level. The analysis at this level was conducted to understand whether the effect observed at the medium level, if any, would relate to the beginning of phonation.

Stepwise multilevel linear models were developed for each stage of the analysis (i.e., stages 1–3), response variable (i.e., absolute asynchronies, signed asynchronies, and SD of absolute asynchronies,), and primary fixed factors (i.e., visual contact and leadership), as shown in Tables 1–**4**. Time category and note were also entered in the model as fixed effects nested in the primary fixed effects, or as random effects, depending on the level of the analysis. Participants were treated as a random variable across levels. At the high level of the analysis, models were designed to test the fixed effects of visual contact, leadership, and time category (the latter nested within the two former), and the random effects of participant and note. At the medium level, models tested the fixed effects of visual contact, leadership, and note subset, i.e., note 1, 3, 19, and 22, (note subset nested within the two former) and the random effect of participants. At the low level, models were developed to investigate the fixed effects of visual contact, leadership, and time category (the latter nested within the two former), and the random effect of participants. Multilevel linear models were chosen because they strengthen the statistical reliability of the fixed effects analyses by providing an evaluation of inter-participant, inter-time category, and inter-note variation (Gelman and Hill, 2007). The models were implemented in R Studio (RStudio, 2015) using the lme4 package.

**Table 1:** Overview of the multilevel linear models developed to investigate the precision of synchronization, with primary effects of visual contact, nested effects of crucial notes and time category, and the random effects of participants and chosen notes.

**Stage of analysis**	**Fixed effect variables**	β **coefficients and significance**			**Random effect variables**	**Row number**
Stage 1: Overall	Visual contact	β(−31.7)^***^, *t*(25000) = −10.5			Participants	1
	Time category nested	VC	NB	n.s.	Chosen notes	2
			NE	β(8.8)^***^, *t*(22587) = 3.3		3
			OF	β(19.4)^***^, *t*(20330) = 5.7		4
		NVC	NB	β(−21.9)^***^, *t*(20893) = −8		5
			NE	β(−21.8)^***^, *t*(22537) = −8.3		6
			OF	n.s.		7
Stage 2: Notes subset	Visual contact	β(−23.9)^***^, *t*(24250) = −7.1			Participants	8
	Crucial notes	VC	3	n.s.		9
			19	β(−20.8)^***^, *t*(4, 247) = −6.2		10
			22	β(−19.3)^***^, *t*(4, 247) = −5.8		11
		NVC	3	β(−27.2)^***^, *t*(4, 247) = −8.2		12
			19	β(−37.6)^***^, *t*(4, 247) = −11.3		13
			22	β(−40.2)^***^, *t*(4, 247) = −12.1		14
Stage 3: Significant note	Visual contact	n.s.			Participants	15
	Time category nested	VC	NE	n.s.		16
		NVC	NE	β(−44.6)^***^, *t*(1, 035.8) = 7.4		17

*n.s., not significant*;

*** *p < 0.001. β – fixed effect coefficient on the predictor being considered – are given above with reference to the specified base level of the factor, i.e., NB, NE or OF vs. the base level ON, and note 3, 19, or 22 vs. the base level note 1*.

The investigation was first conducted at the high level, then the analysis of each response variable proceeded at a medium level when a significant fixed effect was found. Similarly, the analysis moved to the low level if a significant fixed effect was found at medium level. Conversely, if a significant effect was not found at a high or medium level, the analysis was not carried over to a deeper level (i.e., from high to medium, or from medium to low).

A Bonferroni correction was implemented to reduce the possibility of obtaining spurious significant results with multiple multilevel linear models. A *p*-value threshold was set at *p* = 0.0027, based on the assumption that a total of 18 models might have been developed, {3*stages* × 3*responsevariables* × 2*primaryfixedfactors*}, if the analyses proceeded from stages 1–3.

## Results

An initial overview of the full data set is provided in 3.1 by way of descriptive statistics, with the purpose to scrutinize the main characteristics of synchronization in singing ensembles, regardless of condition (i.e., visual contact, and the instruction to act as leader or follower). The remaining two sections (see sections Visual Contact Effect and Effect of the Instruction to Act as Leader or Follower) present the results of the analyses of the main effects of visual contact and the instruction to act as leader or follower on interpersonal synchronization, respectively. β–fixed effect coefficient on the predictor being considered—are given below and in Tables 1–**4** with reference to the specified base level of the factor, i.e., NB, NE, or OF vs. the base level ON, and note 3, 19, or 22 vs. the base level note 1. The β fixed effect coefficients indicate that for each 1 unit increase in the predictor being considered, the effect of the given predictor changes by the amount specified by the β coefficient.

### Synchronization characteristics

**Figure 2:**
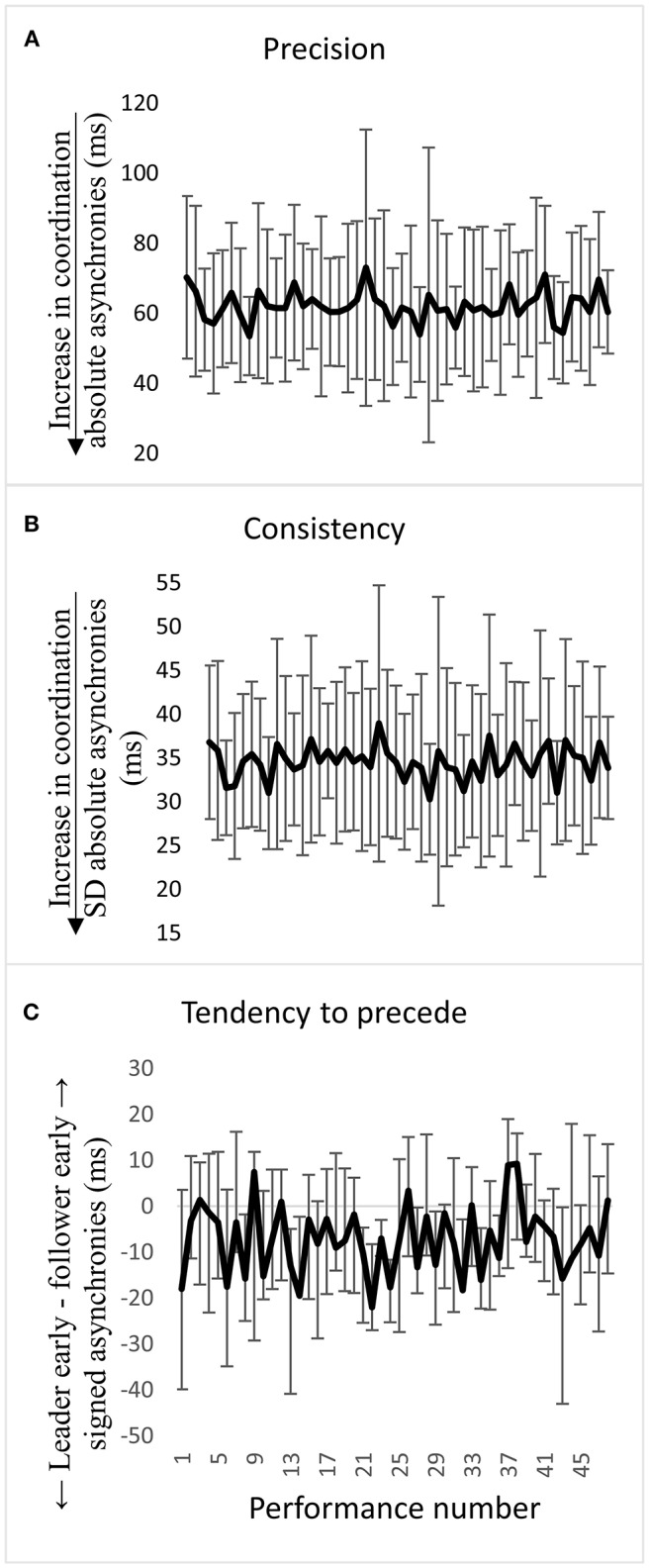
Performance across the 48 trials, indicating precision of synchronization in **(A)** (mean of absolute asynchronies), consistency in **(B)** (SD of absolute asynchronies), and tendency to precede or lag a co-performer in **(C)** (median of signed asynchronies). Error bars of precision and consistency represent standard error of the mean, whilst error bars of the tendency to precede indicate interquartile range of the median.

The analysis of the overall synchronization was computed regardless of performance condition and time categories, taking all notes together and averaging for each duo. Results show that the precision of overall synchronization computed on the mean of absolute asynchronies was on average 58.99 ms (*SD* = 11.13), consistency indexed by SD of absolute asynchronies was 67.06 ms (*SD* = 11.85), whilst the tendency to precede/lag as indexed by the median of signed asynchronies was −4.06 ms (IQR = 4.38). The full sample data were scrutinized to investigate changes in the asynchronies across the course of the 48 repeated performances, by averaging the asynchronies for each measure (median, mean, and SD) and each performance across the 12 duos. Figure 2 represents these data and suggests that, with practice, there was no discernible improvement in synchronization between the singers.

### Visual contact effect

#### Precision

**Figure 3:**
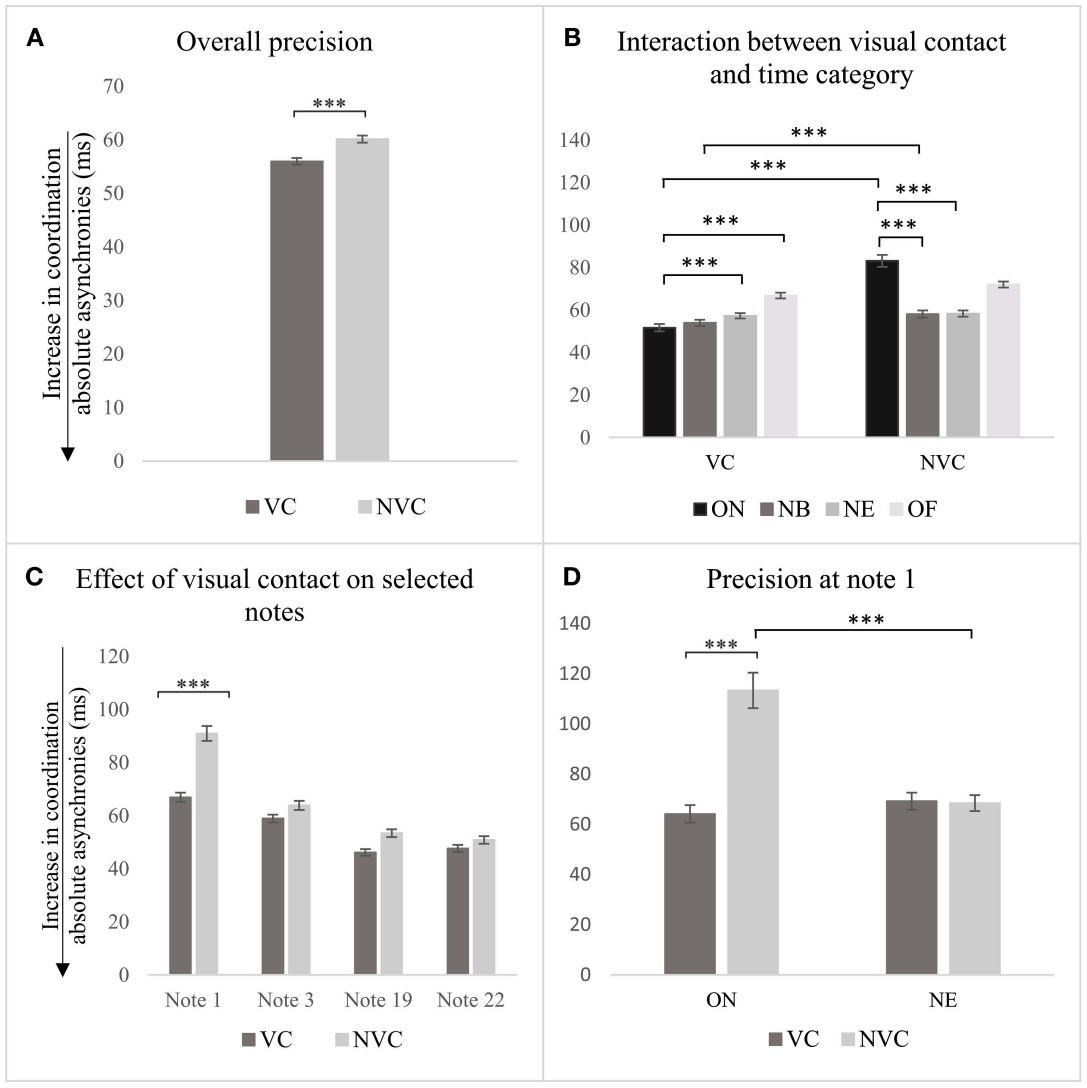
**(A)** Effect of visual contact on precision of synchronization computed overall; **(B)** effect of visual contact in relation to the time categories; **(C)** effect of visual contact computed on a subset of notes, but only comparison between the same pairs of notes is presented; **(D)** effect of visual contact on note 1. Error bars represent 95% CI of the mean. *p*-values have been adjusted using the Holm method. ^***^p < 0.001.

The analysis conducted at stage 1, based on the multilevel linear model developed as explained above, demonstrated that the presence/absence of visual contact between singers predicted precision in the synchronization (see Table 1, row 1), β(−31.7), *t*(25000) = −10.4, *p* < 0.001. As shown in Figure 3A, precision of synchronization was significantly better when visual contact between singers was present, (*M* = 56.0 *ms, SD* = 48.2 *ms*), compared with when visual contact was absent (*M* = 60.1 *ms, SD* = 53.6 *ms*). The variance partition coefficient (VPC) among participants and notes was 0.027 and 0.043, which indicates that only 2.7 and 4.3% of the variability of the effect of visual contact can be attributed to participants and chosen note, respectively. In the presence of visual contact between singers, precision temporally computed at ON was better than that computed at NE β(8.8), *t*(22, 587) = 3.3, *p* < 0.001, and OF β(19.4), *t*(20, 330) = 5.7, *p* < 0.001 (see Table 1, rows 2–4). Interestingly, when visual contact between singers was absent, the relationship between time categories changed: precision computed at ON was lower than that computed at NB, β(−21.9), *t*(20, 893) = −8.0, *p* < 0.001, and NE, β(−21.8), *t*(22, 537) = −8.3, *p* < 0.001(see Figure 3B, and Table 1, rows 5–7). *Post-hoc* tests between same pairs of time categories (e.g., ON in presence and absence of visual contact), calculated with Holm correction for multiple comparisons, show that precision of NB synchronization was significantly better in the presence of visual contact, (*M* = 54.0 *ms, SD* = 48.1), than in its absence, (*M* = 58.0 *ms, SD* = 50.2), *t* = 4.7, *p* < 0.001; likewise, precision in the synchronization computed at ON was better with visual contact between singers, (*M* = 51.7 *ms, SD* = 49.0), than without, (*M* = 83.2 *ms, SD* = 92.0), *t* = 10.5, *p* < 0.001.

When the effect of visual contact was investigated in relation to notes 1, 3, 19, and 22 (i.e., medium level of the analysis), results show that, in the presence of visual contact, precision at note 1 was significantly greater than that computed at note 19, β(−20.8), *t*(4, 247) = −6.2, *p* < 0.001, and note 22 (see Table 1, rows 9–11), β(−19.3), *t*(4, 247) = −5.8, *p* < 0.001. When visual contact was absent, the coefficients of these relationships were even larger: synchronization at note 1 was greater than that at note 3, β(−27.2), *t*(4, 247) = −8.2, *p* < 0.001, note 19, β(−37.6), *t*(4, 247) = −11.3, *p* < 0.001, and note 22, β(−40.2), *t*(4, 247) = −12.1, *p* < 0.001(see Table 1, rows 12-14). The variability of this effect among participants was small (*VPC* = 4.7%). *Post-hoc* comparisons demonstrate that this effect was associated with note 1: precision of synchronization was significantly better with visual contact (*M* = 66.9 *ms, SD* = 55.8), compared to without, (*M* = 90.9 *ms, SD* = 91.3), *t* = 7.139, *p* < 0.001, as shown in Figure 3C.

The analysis conducted at stage 3 highlighted that without visual contact, precision at ON was significantly greater than that at NE (see Table 1, row 17), β(−44.6), *t*(1035.8) = 7.4, *p* < 0.001, and that precision at ON was better with visual contact between singers (*M* = 64.2 ms, *SD* = 56.3), than without (*M* = 113.4 ms, *SD* = 114.7), *t* = 8.0, *p* < 0.001 (see Figure 3D). The variability of this effect among subjects was small (*VPC* = 4.7%).

In summary, these findings show that the presence/absence of visual contact predicted the precision of synchronization, which was better when the visual contact between singers was present, compared with when the visual contact was absent. This effect was constant among participants and was associated with the onset of phonation at the beginning of the piece.

#### Consistency

**Figure 4:**
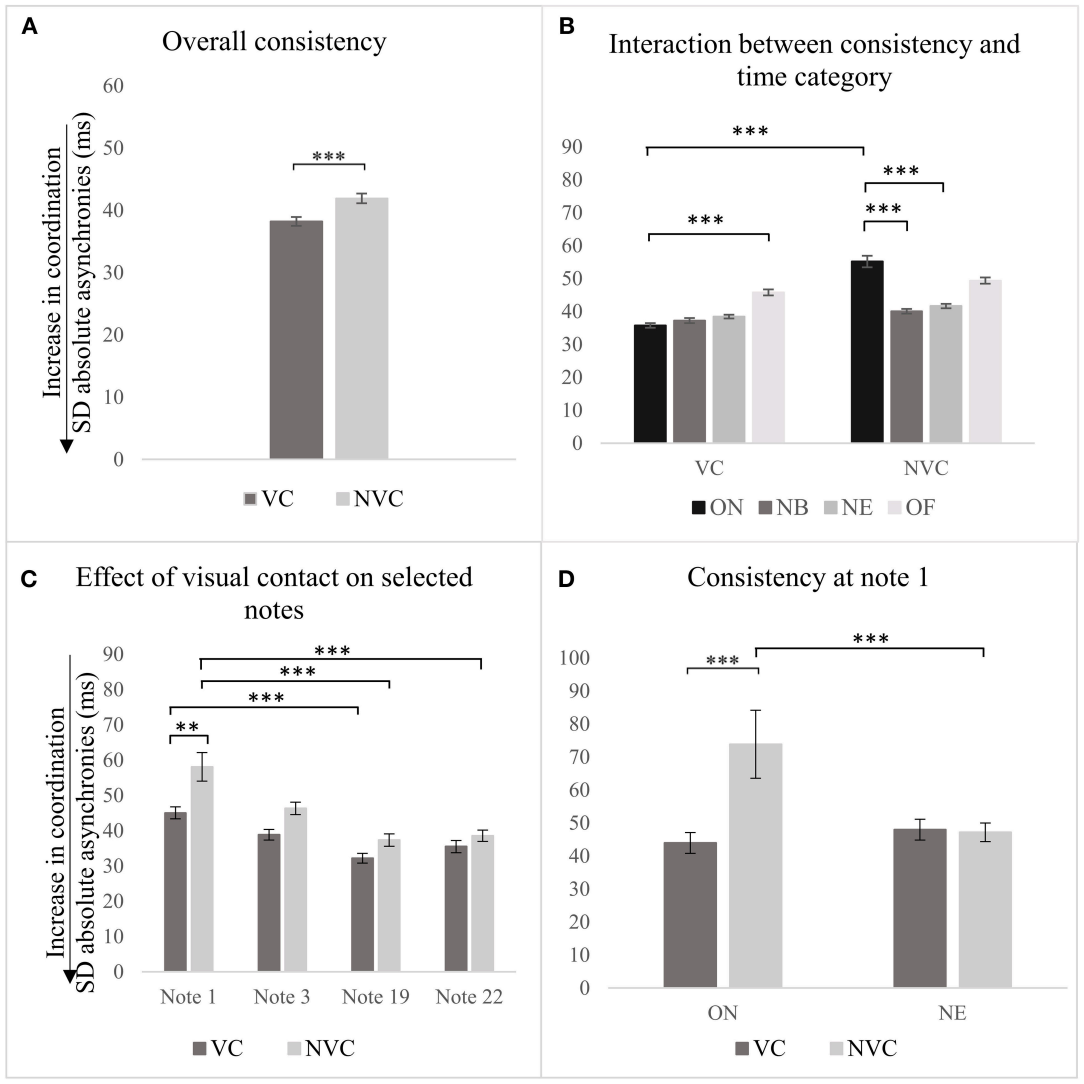
Effect of visual contact on consistency of synchronization, as indexed by SD of absolute asynchronies, computed: for the overall piece **(A)**, in relation to time categories **(B)**, to a subset of notes **(C)**, and to note 1 **(D)**. Error bars represent 95% CI of the mean. *p*-values have been adjusted using the Holm method. ^**^p < 0.01, ^***^p < 0.001.

The analysis conducted at the high level demonstrates that the presence/absence of visual contact predicted the consistency of synchronization as indexed by the SD of absolute asynchronies (see Table 2, row 1), β(−19.6), *t*(2, 224) = −6.1, *p* < 0.001. Synchronization was more consistent with visual contact between singers (*M* = 38.2 *ms, SD* = 17.1) than without (*M* = 41.9 *ms, SD* = 18.7), as shown in Figure 4A. The variability of this effect among participants and chosen notes was small, *VPC* = 9.3% and *VPC* = 14%. With visual contact, synchronization temporally computed at ON was more consistent than that at OF (see Table 2, row 4), β(12.2), *t*(2065.3) = 3.5, *p* < 0.001. But when the visual contact was absent, the relationships between time categories changed again: synchronization computed at ON was less consistent than that at NB, β(−13.8), *t*(2106.8) = −4.8, *p* < 0.001, and NE (see Table 2, rows 5–7), β(−12.5), *t*(2181.6) = −4.5, *p* < 0.001. As highlighted by *post-hoc* testing, Holm corrected for multiple comparisons, synchronization temporally calculated at ON was more consistent in the presence of visual contact, (*M* = 35.8 *ms, SD* = 17.2), than without (*M* = 55.2 *ms, SD* = 41.7), *t* = 6.1, *p* < 0.001, as shown in Figure 4B.

**Table 2:** Overview of the multilevel linear models developed to investigate the consistency of synchronization, with primary effects of visual contact, nested effects of crucial notes and time category, and the random effects of chosen notes and participants.

**Stage of analysis**	**Fixed effect variables**	β **coefficients and significance**			**Random effect variable**	**Row number**
Stage 1: Overall	Visual contact	β(−19.6)^***^, *t*(2, 224) = −6			Participants	1
	Time category nested	VC	NB	n.s.	Chosen notes	2
			NE	n.s.		3
			OF	β(12.3)^***^, *t*(2, 065) = 3.5		4
		NVC	NB	β(−13.8)^***^, *t*(2, 107) = −4.8		5
			NE	β(−12.5)^***^, *t*(2, 182) = −4.5		6
			OF	n.s.		7
Stage 2: Notes subset	Visual contact	β(−13.5)^***^, *t*(370) = −3.4			Participants	8
	Crucial notes	VC	3	n.s.		9
			19	β(−12.7)^**^, *t*(370) = −3.2		10
			22	n.s.		11
		NVC	3	n.s.		12
			19	β(−20.9)^***^, *t*(370) = −5.4		13
			22	β(−19.9)^***^, *t*(370.1) = −3.2		14
Stage 3: Significant note	Visual contact	n.s.			Participants	15
	Time category nested	VC	NE	n.s.		16
		NVC	NE	β(−26.7)^***^, *t*(81) = 3.8		17

*n.s., not significant*;

** *p < 0.01*;

*** *p < 0.001*.

*β–fixed effect coefficient on the predictor being considered – are given above with reference to the specified base level of the factor, i.e., NB, NE or OF vs. the base level ON, and note 3, 19, or 22 vs. the base level note 1*.

Further analysis focussed on notes 1, 3, 19, and 22 (medium level of analysis), demonstrates that in the presence of visual contact, synchronization temporally computed at note 1 was less consistent than that at note 19 (the second simultaneous voice entry of the piece), β(−12.7), *t*(370.1) = −3.2, *p* < 0.01, as shown in Figure 4C and Table 2, row 8. The relationships between this subset of notes were affected by the absence of visual contact between singers: synchronization at note 1 was even less consistent than that at note 19, β(−20.9), *t*(370) = −5.4, *p* < 0.001), and note 22 (see Table 2, rows 13–14), β(−19.9), *t*(370.1) = −3.2, *p* < 0.001. The variability of this effect among participants was *VPC* = 13%. *Post-hoc* comparisons between same pairs of notes in the two different conditions show that this effect relied on the first note of the piece, *t* = −3.4, *p* < 0.05. Synchronization between singers computed at note 1 was more consistent with visual contact between singers, (*M* = 45.0 *ms, SD* = 16.7), than without (*M* = 58.1 *ms, SD* = 40.0).

The analysis focused on the first note of the piece demonstrated that without visual contact, consistency at ON was significantly greater than that at NE (see Table 2, row 17), β(−26.7), *t*(81) = 3.8, *p* < 0.001, and that consistency at ON was better with visual contact between singers (*M* = 43.9 *ms, SD* = 15.6), than without (*M* = 73.8 *ms, SD* = 50.4), *t* = 4.2, *p* < 0.001, (see Figure 4D). The variability of this effect among participants was small, *VPC* = 26.7%.

In summary, the presence and absence of visual contact had a significant effect on the consistency of the temporal coordination of the overall piece: synchronization was more consistent with visual contact between singers, than without it. This effect was consistent among participants and was associated with the synchronization of the onset of the first note of the piece.

#### Tendency to precede or lag a co-performer

**Figure 5:**
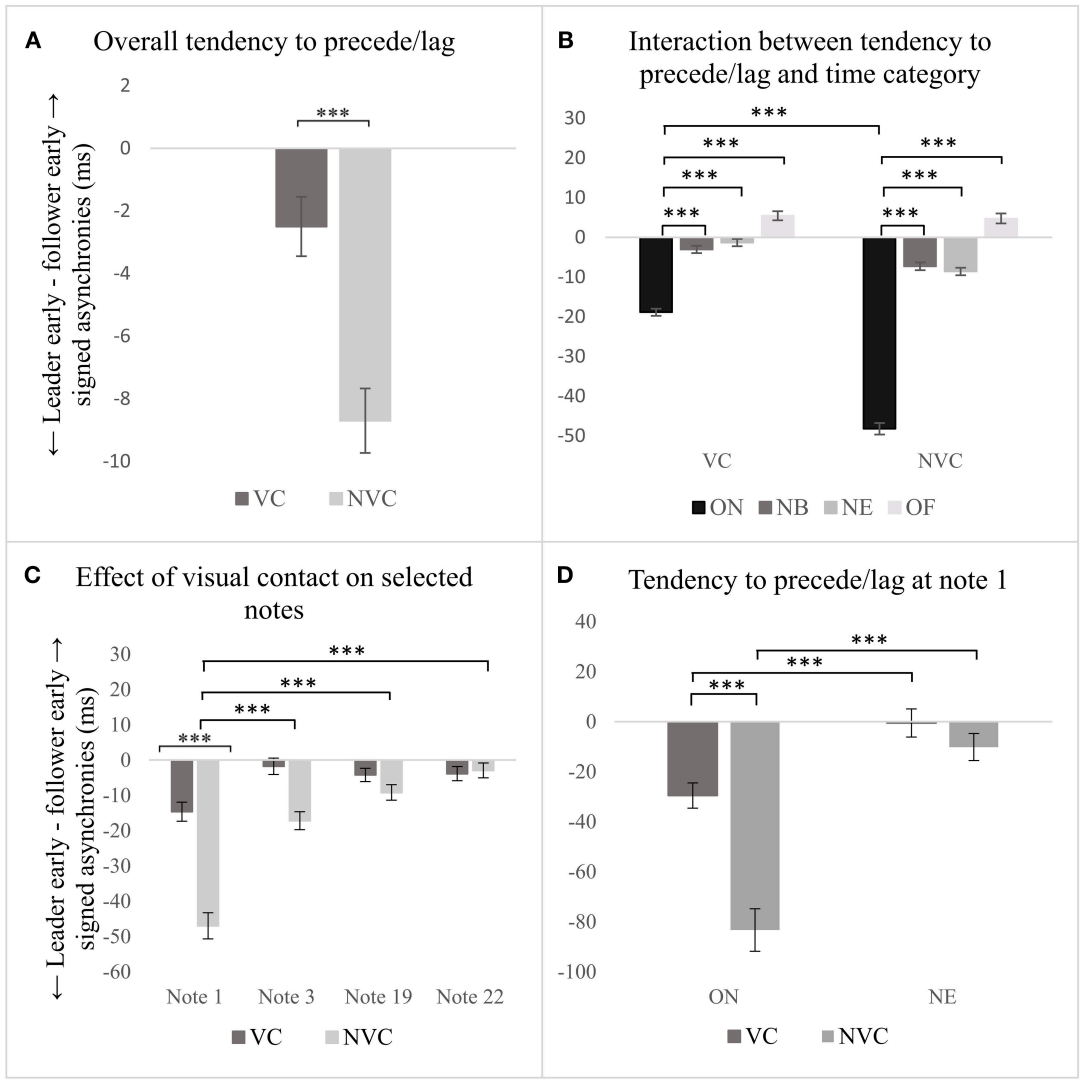
**(A)** effect of visual contact on tendency to precede/lag a co-performer computed overall; **(B)** effect of visual contact in relation to the time category; **(C)** effect of visual contact computed on a subset of notes; **(D)** effect of visual contact on note 1. Error bars represent 95% CI of the mean. *p*-values have been adjusted using the Holm method. ^***^p < 0.001.

The presence/absence of visual contact between singers predicted the tendency to precede or lag a co-performer (see Table 3, row 1), β(28.3), *t*(25.1) = 5.9, *p* < 0.001. The variability of this result attributed to the participants is 0.09%, and the variability among the chosen notes is 0.02%. One sample *t*-tests conducted for difference from 0 show that the designated leader significantly tended to be ahead of the co-performer in the presence of visual contact, *t*(12, 491) = −3.7, *p* < 0.001, as well as without, *t*(12, 661) = −12.0, *p* < 0.001, as shown in Figure 5A. However, the amount by which the leader tended to precede the co-performer without visual contact (*M* = −8.7 *ms, SD* = 81.8) is greater than with (*M* = −2.5 *ms, SD* = 75.1), as highlighted by the fixed-effect coefficient above. In addition, with visual contact, the amount of leading observed at ON was greater than that at NB, β(16.2), *t*(1, 264) = 4.0, *p* < 0.001, NE, β(18.0), *t*(2, 031) = 4.6, *p* < 0.001, and OF, β(23.8), *t*(1, 310) = 4.8, *p* < 0.001. Without visual contact, those relationships are amplified, as highlighted by the following fixed effects coefficients: the amount of leading found at ON was even greater than that observed at NB, β(41.3), *t*(1, 247) = 10.3, *p* < 0.001, NE, β(40.1), *t*(1, 997) = 10.2, *p* < 0.001, and OF, β(52.4), *t*(1, 295) = 10.7, *p* < 0.001. *Post-hoc* testing between the same pairs of time categories, correcting using the Holm method for multiple comparisons, demonstrates that these effects were associated with the tendency to precede/lag a co-performer at ON. The amount of leading computed at ON when visual contact was absent (*M* = −48.2 *ms, SD* = 115.4), was significantly greater than that observed when visual contact was present, (*M* = −18.9 *ms, SD* = 70.3), *t* = −5.9, *p* < 0.001 (see Figure 5B).

**Table 3:** Overview of the multilevel linear models developed to investigate the tendency to lead/lag synchronization, with the primary effects of visual contact, nested effects of crucial notes and time category, and the random effects of participants and chosen notes.

**Stage of analysis**	**Fixed effect variables**	β **coefficients and significance**			**Random effect variable**	**Row number**
Stage 1: Overall	Visual contact	β(28.3)^***^, *t*(25) = 5.9			Participants	1
	Time category nested	VC	NB	β(16.2)^***^, *t*(1, 264) = 4	Chosen notes	2
			NE	β(18)^***^, *t*(2, 031) = 4.6		3
			OF	β(23.8)^***^, *t*(1, 310) = 4.8		4
		NVC	NB	β(41.3)^***^, *t*(1, 247) = 10.3		5
			NE	β(40.1)^***^, *t*(1, 997) = 10.2		6
			OF	β(52.4)^***^, *t*(1, 295) = 10.7		7
Stage 2: Notes subset	Visual contact	β(32.1)^***^, *t*(4270) = −1			Participants	8
	Crucial notes	VC	3	n.s.		9
			19	n.s.		10
			22	n.s.		11
		NVC	3	β(29.8)^***^, *t*(4, 261) = 6		12
			19	β(37.8)^***^, *t*(4, 261) = 7.6		13
			22	β(44.1)^***^, *t*(4, 261) = 8.9		14
Stage 3: Significant note	Visual contact	n.s.			Participants	15
	Time category nested	VC	NE	β(29.1)^***^, *t*(1049.8) = 3.4		16
		NVC	NE	β(73)^***^, *t*(1049.9) = 8.5		17

*n.s. = not significant*;

*** *p < 0.001*.

*β–fixed effect coefficient on the predictor being considered–are given above with reference to the specified base level of the factor, i.e., NB, NE, or OF vs. the base level ON, and note 3, 19, or 22 vs. the base level note 1*.

The analysis of the tendency to precede/lag a co-performer in the presence of visual contact demonstrated that the subset of notes was not a predictor of the tendency to precede/lag (see Table 3, rows 9–11). Conversely, when visual contact was absent, the amount of leading observed at note 1 was significantly greater than that computed at note 3, β(29.8), *t*(4, 261) = 6.0, *p* < 0.001, note 19, β(37.8), *t*(4, 261) = 7.6, *p* < 0.001, and note 22, β(44.1), *t*(4, 261) = 8.9, *p* < 0.001, as shown in Figure 5C and Table 3, rows 12–14. The variability of this effect among participants was small (*VPC* = 1.3%). *Post-hoc* comparisons between same pairs of notes in the two different conditions demonstrate that the effect of presence/absence of visual contact between singers is associated with the synchronization of note 1; the amount of leading was significantly greater when visual contact was absent, (*M* = −47.0 *ms, SD* = 121.3), compared with when visual contact was present, (*M* = −14.7 *ms, SD* = 88.9), *t* = −6.4, *p* < .001.

The analysis of the first note of the piece demonstrated that the time category predicted the tendency to precede/lag a co-performer in both conditions, i.e. with and without visual contact (see Table 3, rows 16–17). However, the amount of leading was greater in the absence of visual contact, as highlighted by the fixed-effect coefficients: the tendency to precede/lag the co-performer at ON was larger than that at NE in the presence of visual contact, β(29.1), *t*(1049.8) = 3.4, *p* < 0.001; but, the leading observed at ON was even greater than that at NE without visual contact, β(73), *t*(1049.9) = 8.5, *p* < 0.001. *Post-hoc* testing highlighted that these effects were associated with the ON: the tendency to precede/lag the co-performer was greater when visual contact was absent (*M* = −83.3 *ms, SD* = 138.3), compared with when visual contact was kept (*M* = −29.6 *ms, SD* = 82.7), *t* = −6.2, *p* < 0.001 (see Figure 5D). The variability among participants of the effect of visual contact on the tendency to precede/lag note 1 was small (*VRP* = 7.2%).

In summary, these results demonstrate that without visual contact, the designated leader showed overall a stronger tendency to precede the designated follower, than with visual contact. This effect was consistent among participants and was associated with a stronger tendency to precede the designated follower at the onset of note 1 when no visual contact was available.

### Effect of the instruction to act as leader or follower

#### Precision and consistency

**Figure 6:**
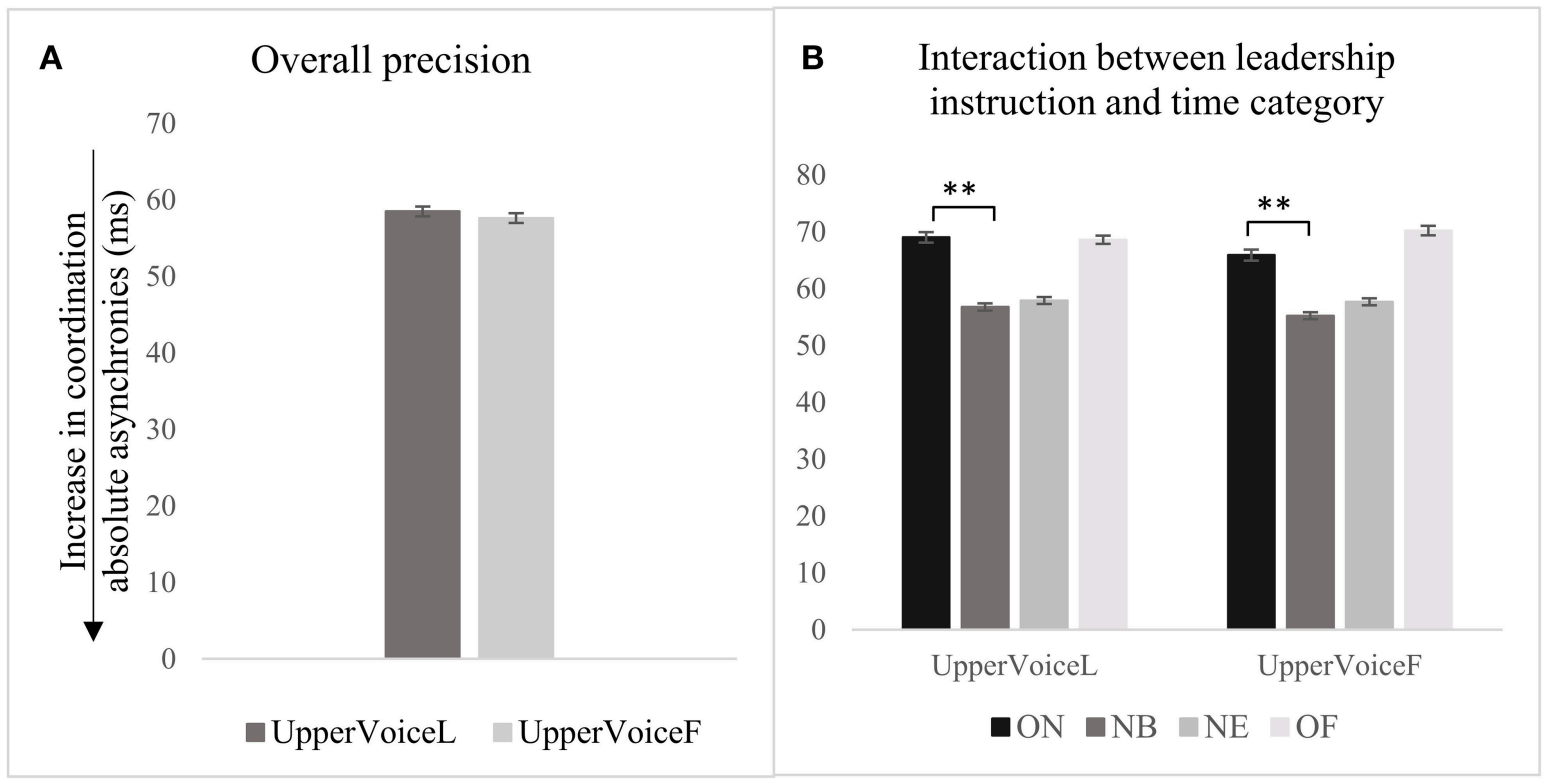
Effect of instruction to the precision of synchronization computed for the whole piece **(A)**, and in relation to the time categories **(B)**. Error bars represent 95% CI of the mean. *p*-values have been adjusted using the Holm method. ^**^p < 0.01.

The instruction to act as leader or follower of the performance did not predict precision of the synchronization of the whole piece, as shown in Figure 6A and Table 4, row 1. This result did not vary greatly among participants (*VPC* = 2.6%) or note (*VPC* = 4.3%). Precision at ON was significantly greater compared with NB when the upper voice was instructed to lead, β(−8.9), *t*(20, 806) = −3.2, *p* < 0.01, and also when instructed to follow β(−7.4), *t*(20, 926) = −2.7, *p* < 0.01 (see Figure 6B and Table 4, rows 2 and 5). When the upper voice was leading, for each unit of increase in the precision computed at onsets, precision at note beginnings decreased by 8.9 ms; when the upper voice was lagging, precision at note beginnings decreased by 7.4 ms. *Post-hoc* tests did not show a significant difference between same pairs of time categories in the two different conditions (i.e., when upper voice was instructed to lead or follow). Since the leadership instruction was not a significant predictor of precision at stage 1, the analysis was not conducted for deeper levels, i.e., stages 2 and 3.

**Table 4:** Overview of the multilevel linear models developed to investigate the precision and consistency of synchronization and the tendency to lead/lag, with the primary effects of leadership instruction, nested effects of crucial notes and time category, and the random effects of participants and chosen notes.

**Synchronization parameter**	**Stage of analysis**	**Fixed effect variables**	β **coefficients and significance**			**Random effect variables**	**Row number**
Precision	Stage 1: Overall	Leadership	n.s.			Participants	1
		Time category nested	UVL	NB	β(−8.9)^**^, *t*(20, 806) = −3.2	Chosen notes	2
				NE	n.s.		3
				OF	n.s.		4
			UVF	NB	β(−7.4)^**^, *t*(20, 926) = −2.7		5
				NE	n.s.		6
				OF	n.s.		7
Consistency	Stage 1: Overall	Leadership	n.s.			Participants	8
		Time category nested	n.s.			Chosen notes	9
Tendency to	Stage 1: Overall	Leadership	n.s.			Participants	10
lead		Time category nested	UVL	NB	β(26.2)^***^, *t*(1247) = 6.6	Chosen notes	11
				NE	β(32.3)^***^, *t*(1, 996) = 9.5		12
				OF	β(43.9)^***^, *t*(1, 286) = 9		13
			UVF	NB	β(31.5)^***^, *t*(1, 271) = 7.9		14
				NE	β(20.8)^***^, *t*(2, 044) = 5.3		15
				OF	β(32.4)^***^, *t*(1, 327) = 6.6		16

*n.s., not significant*;

** *p < 0.01*;

*** *p < 0.001*.

*β – fixed effect coefficient on the predictor being considered - are given above with reference to the specified base level of the factor, i.e., NB, NE, or OF vs. the base level ON*.

**Figure 7:**
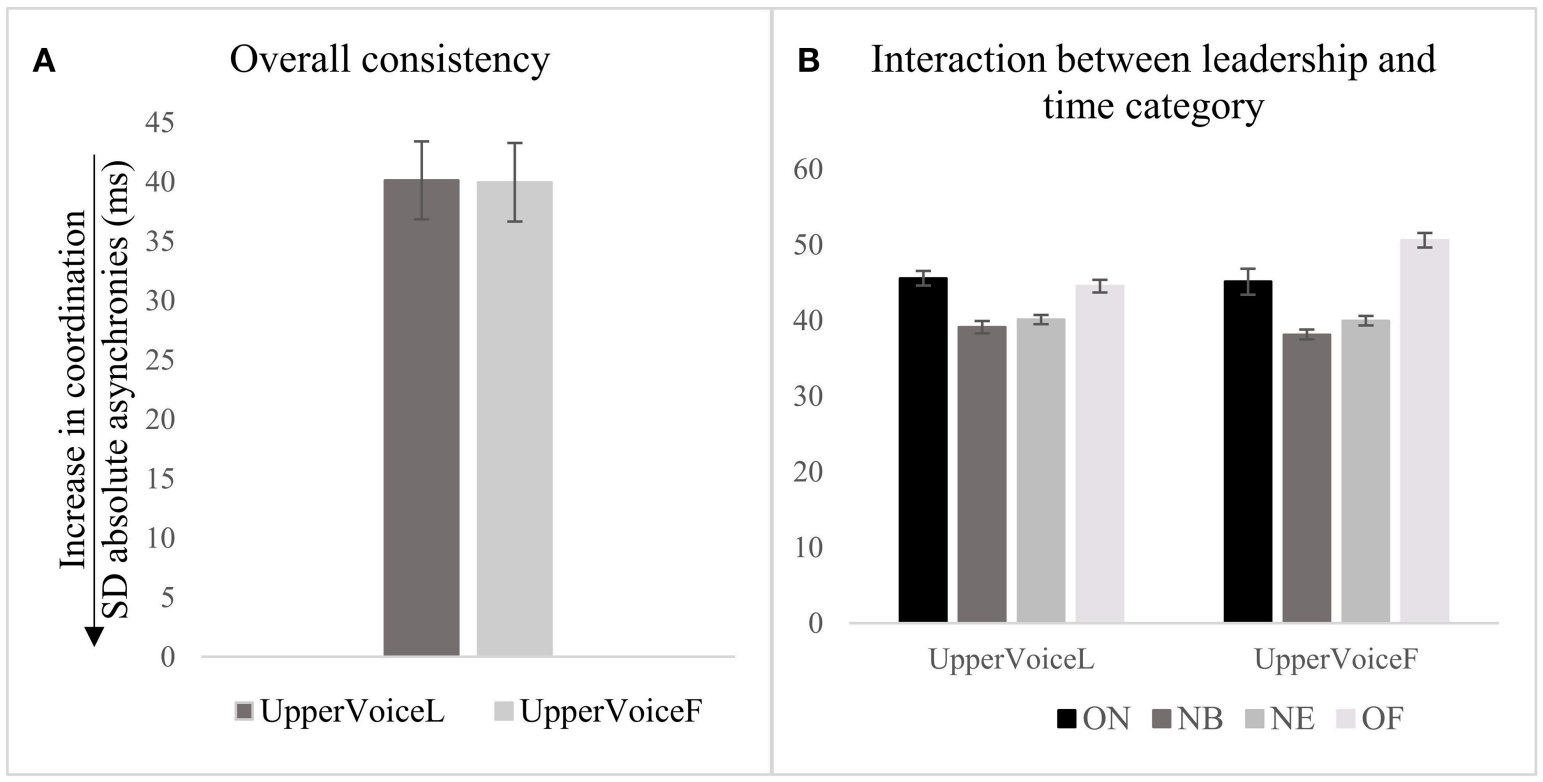
Instruction to act as leader or follower and the consistency of synchronization as indexed by SD of absolute asynchronies, computed for the overall piece **(A)**, and in relation to time categories **(B)**.

As shown in Figure 7A and Table 4, row 8, the effects of instruction on the consistency of synchronization as indexed by the SD of absolute asynchronies were not found, and the variability of these results was small among participants (for SD asynchronies, *VPC* = 9.1%) and notes (for SD asynchronies, *VPC* = 13.6%). The instruction was not associated with differences between ON and NB, ON and NE, or ON and OF (see Figure 7B and Table 4, row 9).

#### Tendency to precede or lag a co-performer

**Figure 8:**
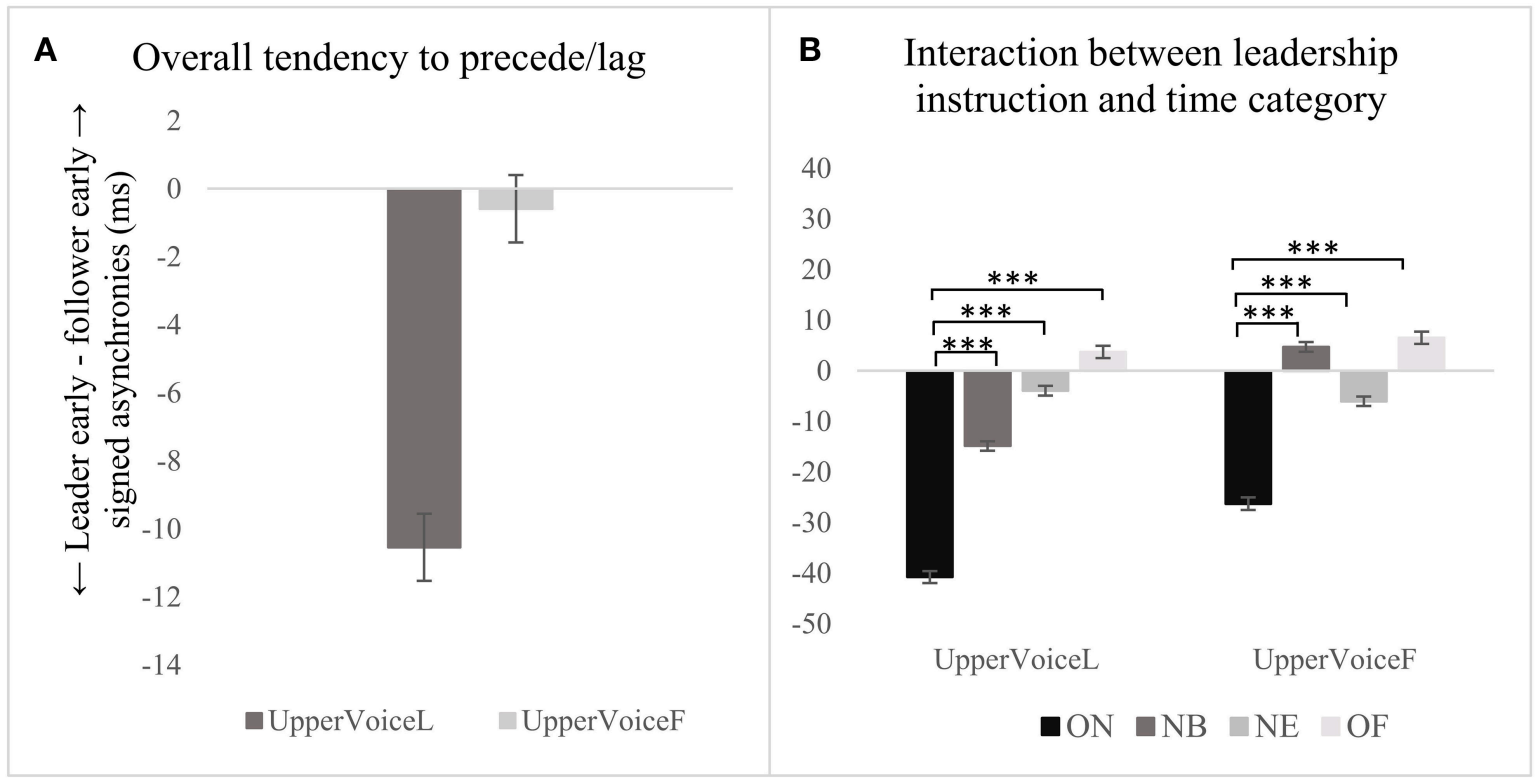
Instruction to act as leader or follower and the tendency to precede/lag a co-performer **(A)**, and relationships between time categories **(B)**. ***p < 0.001.

The analysis conducted at stage 1 shows that the instruction to act as leader or follower of the performance did not predict the tendency to precede/lag a co-performer (see Figure 8A and Table 4, row 10); the variability of this effect among participants was 0.1% and chosen notes was 0.2%. One sample *t*-tests conducted for difference from 0 show that the designated leader significantly tended to be ahead of the co-performer when the upper voice was instructed to lead, *M* = −10.5 *ms, SD* = 78.1, *t*(12, 491) = −3.7, *p* < 0.001. When the upper voice was instructed to follow, nobody tended to precede/lag the co-performer overall. In addition, the tendency to precede/lag changes according to the time category regardless of the instruction (see Figure 8B). When the upper voice was instructed to lead, the degree of leading observed at ON was greater than that found at NB, β(26.2), *t*(1, 247) = 6.6, *p* < 0.001, NE, β(32.3), *t*(1, 996) = 9.5, *p* < 0.001, and OF, β(43.9), *t*(1286) = 9.1, *p* < 0.001 (see Table 4, rows 11–13). Similarly, when the upper voice was instructed to follow, the amount of leading by the lower voice observed at ON was greater than that found at NB, [β(31.5), *t*(1271) = 7.9, *p* < 0.001], NE [β(20.8), *t*(2, 044) = 5.3, *p* < 0.001], and OF [β(32.4), *t*(1, 327) = 6.6, *p* < 0.001] (see Table 4, rows 14–16).

Since the results did not show a significant effect of the leadership instruction on the tendency to precede/lag a co-performer, the analysis was not conducted at a deeper level.

## Discussion

This study investigated whether visual contact and assigned leadership roles contribute to interpersonal synchronization during singing duo performances. Three measures of interpersonal synchronization were considered: precision of synchronization as quantified by absolute asynchronies, consistency represented by SD of absolute asynchronies, and the tendency to precede or lag a co-performer indicated by signed asynchronies.

The presence or absence of visual contact between singers had a significant effect on the precision and consistency of synchronization, being better when the visual contact between singers was present, compared with when the visual contact was absent. In comparison, the results reported in the pilot study conducted by D'Amario et al. (2018) with two singing duos have shown an increase in the consistency and precision of synchronization when there was no visual contact between singers in the case of duo 1 and duo 2, respectively. These apparent different results can be understood in light of the different sample size. Visual contact also had an effect on the tendency to precede or lag a co-performer: without visual contact, the designated leader showed overall a stronger tendency to precede the designated follower than in the presence of visual contact. These effects were consistent across participants and notes. These results expand on previous research focused on the effect of visual contact on instrumental ensembles and suggesting that visual contact might affect synchronization during instrumental performances when auditory feedback is limited (Goebl and Palmer, 2009) and in the presence of tempo changes (Kawase, 2014; Bishop and Goebl, 2015). The present study shows that visual contact might affect interpersonal synchronization also during singing duo performances. In addition, this study builds on previous investigations analyzing interpersonal synchronization during ensemble performances, in which the tempo was controlled by a metronome, and musicians were clearly required to focus on timekeeping (Goebl and Palmer, 2009; Keller and Appel, 2010). This study contributes to knowledge of the role of visual contact in interpersonal synchronization, as emerging spontaneously during repeated performances rather than being forced by a metronome.

In addition, the results demonstrate that effects of visual contact on aspects of synchronization were seen most strongly at the onset of the first note. Precision and consistency observed at the onset of note 1 were better with visual contact, compared to when visual contact was absent. The tendency to precede the co-performer at the onset of note 1 was stronger when visual contact was absent than when the singers could see each other. These results show that visual contact might affect the synchronization of the onset of the first note, but musicians are able to compensate soon after, achieving a tighter interpersonal coordination, which also suggests optimal feedback adaptation. These findings expand on D'Amario et al. (2018) who found that visual contact affected synchronization temporally computed at note beginnings, but not at note endings. These findings are particularly beneficial for the identification of strategies to improve ensemble music performance, refining rehearsal techniques and improving the experience of ensemble singing across all abilities.

When performed with visual contact, precision and consistency computed at the beginning of phonation of the piece were different than those computed at the onset of note 19 (another simultaneous entry point) and beginning of note 22. These differences were amplified when performed without visual contact. The tendency to lead/lag at the beginning of phonation of the piece was not different from that computed at other onsets of the piece (i.e., note 19) or other note beginnings (i.e., note 3 and 22) with visual contact. However, without visual contact, the amount of leading at the onset of note 1 was greater than that computed at other onsets (i.e., note 19) and note beginnings (i.e., note 3 and 22). These results suggest that synchronization computed at the onset of note 1 might be different than other onsets of the piece, and note beginnings within a legato phrase. These differences might be intensified when visual contact is absent.

The researcher's instruction to act as leader or follower of the performance had no overall effect on the precision and consistency of synchronization, or tendency to lead or lag a co-performer. When the upper voice was instructed to lead, the designated leader tended to precede the follower by a small, but significant amount. Notably, when the upper voice was the designated follower, there was no clear separation of roles. These findings are consistent across participants and notes. These results complement the findings reported by Goebl and Palmer (2009) for piano duets performing melody-accompaniment pieces, and by Zamm et al. (2015) analyzing piano duets performing the same part in unison and round. Overall, the results suggest that the effect of the instruction to lead or follow might depend on the piece being performed. The designated leader is more likely (i) to precede the performance of onsets in melody-accompaniment pieces (Goebl and Palmer, 2009); (ii) to lag the performance of the onsets when participants performed the same parts in a round (Zamm et al., 2015); and, (iii) to not be affected by the instruction to act as leader or follower when performing a two-part piece with a less clear separation of roles induced by the score, as found in this study. The last finding suggests that trained musicians might have developed a compensatory behavior, enabling them to maintain a tight and consistent synchronization, regardless of who is the leader or follower.

Precision at ON was significantly larger compared with NB, when the upper voice was instructed to lead and also when instructed to follow, suggesting that precision at the beginning of phonation is larger than that at NB. Instructing the upper voice to lead appears to have intensified the difference in the precision of synchronization between ON and NB. The tendency to lead/lag was different based on the time category considered in relation to the leadership instruction, suggesting a bidirectional adaptation rather than a clear adaptation of roles. This finding corroborates the recent case study conducted among two singing duos by D'Amario et al. (2018), suggesting reciprocal adaptations between musicians that are not limited to the attack of the note, but associated also with note beginnings, endings and offsets.

## Limitations and future work

In this study, the tendency to precede or lag timing is considered an indicator of the leader-follower relationships between the singers, as is common in this field of research (Goebl and Palmer, 2009; Keller and Appel, 2010; Palmer et al., 2013; Timmers et al., 2013, 2014; Zamm et al., 2015). Nevertheless, the fact that one of the musicians might tend to anticipate or lag each other is not a comprehensive perspective on leadership, which can be viewed more in terms of social roles than in terms of performance timing. The analysis of leader-follower relationships based on the combined analysis of synchronization during ensemble performances and patterns of social interactions emerging during rehearsals and investigated through the study of patterns in verbal behaviors, rehearsal tasks and methods, is currently under investigation to shed more light on our understanding of leader-follower relationships in singing ensembles.

Another avenue for consideration is the investigation of the perceptibility of the effects of altered visual contact for listeners with different levels of musical expertise. Previous studies suggest that listeners are sensitive to the degree of between-player asynchrony, when judging lack of togetherness in string quartet performances (Wing et al., 2014a), and that musicians show greater perceptual sensitivity to timing variability than non-experts during isochronous auditory tasks (Repp, 2010). However, whether a listener could detect differences in asynchronies of recordings performed with or without visual contact between musicians has not yet been investigated, to the best of our knowledge. An investigation has been planned of the perception of synchronization during singing duo and quintet performances by participants with varying levels of musical and performance expertise.

This study concerned semi-professional singing duos, performing a short, mostly homophonic piece. To understand whether the above effects typify the ensemble and/or the music piece being performed, it will be necessary for future studies to build a corpus of research which will gradually examine the consistency of the above results across performances of different excerpts, and type and size of ensemble. The experiment should also be replicated with professional singers, since some synchronization patterns might change according to the musicians' level of expertise.

## Conclusions

This study assessed the impact of visual contact and leader and follower relationships on the synchronization of singing duos. Results show that the presence and absence of visual contact between singers had a significant effect on the precision and consistency of interpersonal synchronization, and on the tendency to lead or lag a co-performer during vocal duo performances. Precision and consistency were better when the singers could see each other than when they could not. The tendency to precede or lag a co-performer was greater without visual contact, and this effect was associated with the onset of note 1. These findings were consistent across performers. The instruction to act as leader or follower of the performance did not affect the precision and consistency of interpersonal synchronization, nor the tendency to precede or lag a co-performer. The variability of these results among singers was small.

Synchronization is likely to change based on the time category considered, being often larger at the onset of phonation at the beginning of the piece. The absence of visual contact and instructing the upper voice to lead is likely to amplify differences between time categories.

This study provides a novel contribution to research in this area by investigating synchronization in ensemble singing, an area that has received very little attention to date.

In addition to highlighting valuable avenues for further investigation, these findings could contribute to the tailoring of rehearsal techniques and performance practice to improve synchronization in singing ensembles. This study contributes also to the investigation of the psychological processes that underlie human interpersonal communication and social interaction, identifying the role of visual contact and leader-follower relationships between musicians during singing ensemble performances.

## Author contributions

SD was the principal investigator and made substantial contributions to the conception and design of the study, data acquisition, analysis and interpretation. She drafted the article and approved the submitted version. HD contributed to the design of the study and critically revised the article and approved the submitted version. FB made a useful contribution to the analysis of the data collected, and critically revised the manuscript and approved the submitted version.

### Conflict of interest statement

The authors declare that the research was conducted in the absence of any commercial or financial relationships that could be construed as a potential conflict of interest.
